# Evaluation of an equine-assisted therapy program for veterans who identify as ‘wounded, injured or ill’ and their partners

**DOI:** 10.1371/journal.pone.0203943

**Published:** 2018-09-27

**Authors:** Madeline Romaniuk, Justine Evans, Chloe Kidd

**Affiliations:** 1 Gallipoli Medical Research Institute, Veteran Mental Health Initiative, Greenslopes Private Hospital, Brisbane, Queensland, Australia; 2 Faculty of Health and Behavioural Sciences, The University of Queensland, Brisbane, Queensland, Australia; 3 Institute of Health & Biomedical Innovation, Queensland University of Technology, Brisbane, Queensland, Australia; 4 Institute of Resilient Regions, University of Southern Queensland, Springfield Central, Queensland, Australia; University of California, San Francisco, UNITED STATES

## Abstract

The aim of this study was to evaluate outcomes of an equine-assisted therapy program for Defence Force veterans and their partners across the psychological domains of depression, anxiety, stress, posttraumatic stress, happiness, and quality of life, as well as compare the outcomes of an Individual and Couples program. A non-controlled, within-subjects longitudinal design was utilized with assessment at three time points (pre-intervention, post-intervention, and three months follow-up). Between-subjects analysis with two groups was also conducted to compare the outcomes of the Individual and Couples programs. Participants were recruited from ten programs in 2016 with a total of 47 veterans and partners from both an Individual program (*n* = 25; veterans only) and a Couples program (*n* = 22). Outcome measures included the Depression Anxiety Stress Scale-21, Posttraumatic Stress Disorder Checklist for DSM-5, Oxford Happiness Questionnaire, and Quality-of-Life Enjoyment and Satisfaction Questionnaire-Short Form. Paired samples *t*-tests revealed that within both the Individual and Couples programs, there were significantly fewer psychological symptoms and significantly greater levels of happiness and quality of life at post-intervention compared to pre-intervention. Reduced psychological symptoms were maintained at the three months follow-up for participants of the Couples program only. Independent samples *t*-tests revealed participants in the Couples program reported significantly less symptoms of depression, stress, and posttraumatic stress disorder (PTSD) at follow-up compared to participants in the Individual program. These results indicate there may only be meaningful benefits for equine-assisted therapy in the reduction of depression, stress, and PTSD symptoms for veterans, if partners are integrated into the intervention.

## Introduction

Extensive research has highlighted substantial rates of psychiatric disorders as well as symptoms of posttraumatic stress disorder (PTSD), depressive and anxiety disorders among Australian military veterans [1–5]. Research has also noted that partners of military veterans with psychiatric disorders are vulnerable to increased psychological distress and occurrences of psychiatric disorders themselves [6–9]. Poor mental health is associated with substance abuse [10], suicidal ideation and behaviours [7, 11], physical health comorbidities [12, 13], and reduced quality of life [1]. Given the elevated rates of disorders in this population, research into effective psychological treatments is vital. PTSD in particular can be a complex condition to treat and there remains evidence of dropout and non-response rates of up to 50% in studies of empirically supported treatments [14]. Within the veteran population there are also additional barriers to seeking traditional psychotherapy for psychological conditions. One of the most dominant barriers noted in past research is the stigma that is associated with mental health disorders and accessing psychotherapeutic treatment [15–18]. In an attempt to mitigate the perception of stigma with mental health services, there has been growing interest in the utility of ‘adjunct’ therapy interventions which are not considered the first line treatment for PTSD or commonly co-occurring conditions, but which can be considered a part of the battery of interventions that may be useful in management of mental health symptoms for this population. An adjunct therapeutic intervention that has recently gained a following in the veteran community is equine-assisted therapy.

### Equine-assisted interventions

Worldwide, there are now more than 600 equine-assisted therapy programs designed for patients with a broad range of psychological and physical conditions [19]. Equine-assisted therapy is an adjunct intervention that incorporates experiential activities with horses within a traditional therapeutic framework (such as cognitive behavioural therapy or relational Gestalt therapy) to treat a range of psychiatric symptoms and disorders [20, 21]. Research has assessed the effectiveness of equine-assisted therapy programs [21–31], equine-assisted activities [32, 33] as well as therapeutic horse riding [34, 35, 36] aimed at reducing psychological symptoms. Participants of these programs have reported reduced anxiety and depression symptoms [22, 29, 32, 33] reduced PTSD symptoms [34, 35], elevated self-esteem and self-awareness [30], improved communication and trust [31, 34], and increased overall well-being [23]. However, the majority of this research has focused on children and adolescents [24, 31, 32] with limited peer-reviewed studies investigating the utility of equine-assisted therapy for military veterans [25, 27, 33].

Lanning and Krenek [33] conducted an exploratory, mixed methods study investigating the outcomes of an equine-assisted activities program for 13 veteran participants. Prior to participation in the program, qualitative analysis revealed common themes of “hopelessness”, “need for healing”, “isolation”, and “depression” as descriptors of the participants’ current state. When the participants were asked to reflect on the changes they experienced as a result of the intervention, themes of “increased sociability”, “reduced feelings of isolation”, “increased sense of trust and hope”, and “increased need to serve others” were noted [33]. Quantitative data indicated that participants who completed the 12 sessions of the program reported less physical and emotional limitations in a number of health domains. Participants also experienced a reduction in depression symptoms over time, as measured by the Beck Depression Inventory (BDI-II). However no statistical analysis was conducted on this data to determine if this drop in symptoms was significant [33].

Results from an evaluation of a series of pilot workshops of an equine-assisted learning program demonstrated positive outcomes for veterans diagnosed with PTSD [25]. The pilot study (*n* = 31 veterans; *n* = 25 spouses/partners) utilized a self-report measure developed by the researchers (yet to be validated), which included two sub-scales: Relieving Symptoms of PTSD subscale and Acquisition of Coping Skills subscale [25]. The study found 87.1% of veteran participants reported “very positive perceived benefit” regarding relief from their PTSD symptoms. They also stated 100% of the veterans sample reported “very positive perceived benefit” with respect to acquiring new or enhanced self-mediation coping skills [30]. In terms of the spouses/partner’s experience, 88% of partners rated their veteran spouses/partners as having had a reduction with respect to PTSD symptoms. In addition, 92% of spouses/partners rated the veterans “very positive” for acquiring the coping skills necessary to improve their personal relationships [25].

Recently, Ferruolo [27] described the qualitative outcomes of an equine-assisted psychotherapy pilot program for homeless and unemployed veterans living at a Veterans’ Affairs hospital (*n* = 8). The program was conducted through an established therapeutic horse farm and facilitators were masters-level trained social work and counselling professionals. The two-day program consisted of four segments including psycho-education, guided experiential equine activities, group processing, and personal reflection. Overall, participants’ qualitative responses about the perceived benefits of the program included “learning about self”, “spiritual connection”, “trust”, and “respect” [27].

Based on the available literature, there are limited conclusions that can be drawn regarding the efficacy of equine-assisted activities and therapy programs as an adjunct treatment for psychological conditions and associated symptoms among veterans. While demonstrating some promising trends, the studies outlined above include a number of limitations such as a large variation in methodological approach, absence of follow-up data, and limited use of quantitative and standardised psychometric assessment tools.

### The current study

The current study seeks to expand on prior research and contribute to the growing evidence regarding the utility of equine-assisted therapy programs for veteran populations. The aim is to evaluate the outcomes of an equine-assisted therapy program for veterans who identify as ‘wounded, injured, or ill’ and their partners across the psychological domains of posttraumatic stress, depression, anxiety, stress, happiness, and quality of life, as well as compare the outcomes of an Individual and Couples program using standardised psychometric questionnaires and longitudinal follow-up.

## Method

### Design

The study utilized a non-controlled within-subjects longitudinal design. Between-subjects analysis with two groups was also conducted to compare the outcomes of the Individual and Couples programs. As the study is an evaluation of an existing program offered within a Veterans Service Organisation there was no random allocation to a control group condition or a naturally occurring control group available for comparison. Psychometric assessment measures were completed prior to starting the equine-assisted therapy program (pre-intervention), on conclusion of the program (post-intervention), and three months following the conclusion of the program (follow-up). Between the post-intervention and follow-up time points, participants continued treatment as usual and did not engage in further equine-assisted therapy or equine activities.

### Recruitment of participants

Ethics approval was obtained from the Australian Department of Veterans’ Affairs Human Research Ethics Committee (EO16/005). Participants were members of a Veterans Service Organisation, Mates4Mates, who identify as wounded, injured, or ill and completed the equine-assisted therapy program in the 2016 calendar year. Potential participants were referred to the study by staff at the organisation during the enrolment process. Participants were informed of the purpose and aims of the study verbally by staff and through the provision of the Participant Information Sheet. They were advised that if they declined to participate in the study, it would not affect their enrolment in the program. Written informed consent was obtained prior to any study-specific procedures or assessments. The inclusion criteria for eligibility to participate in the study were: (1) ex-serving Defence Force personnel or partners of ex-serving Defence Force personnel; (2) member of the Veterans Service Organisation, Mates4Mates; and (3) approved to complete the program by a Mates4Mates’ psychologist. There were no additional study exclusion criteria. Membership of Mates4Mates is on the condition that you served/are serving in the Australian Defence Force (ADF) and you identify as ‘wounded, injured or ill’ which includes self-report of a physical and/or psychological condition.

### Materials and measures

#### Equine-assisted therapy program

Equine Encounters Australia (EEA) was engaged by Mates4Mates as their provider of equine-assisted therapy courses. The equine-assisted therapy program is a live-in residential therapy course held over a period of five days. The programs are led by two EEA facilitators, certified in the Equine Psychotherapy Institute (Australia) Program Model of Equine Assisted Learning/Psychotherapy and accompanied by a registered psychologist who assists in group work throughout the program. Based on the Equine Psychotherapy Institute (Australia) Model of Equine-Assisted Learning/Psychotherapy [28], the program also incorporates Relational Gestalt Therapy, mindfulness, grounding techniques, and elements of natural horsemanship. All sessional work is experiential and based around learning new skills in order to create social engagement. The therapeutic work is integrated throughout the activities by inviting participants to notice and explore their issues, challenges, and behaviours, and build awareness of their responses (e.g., fear, anxiety, and anger). As the work is based on Relational Gestalt Therapy, participants are never asked to recount or revisit their past experiences; instead, all phenomenological enquiry and observations are kept in the present. Additionally, group discussions provide participants with the opportunity to process and reflect on their experiences from the activities and the day and allows enquiry and observation from the facilitation team. The program does not include learning to ride. All programs are run from working horse properties which have outside arenas, small yards and areas for trails. All properties are rurally based and range in size from 40 to 300 acres.

The programs are run in an ‘Individual’ and ‘Couples’ format, with the latter including partners of veterans. The Couples program includes the same therapeutic activities as the Individual program but also incorporates couples dates and couples counselling to improve communication skills, build trust and respect, and develop shared and individual future goals. See Table 1 for a detailed overview of program content. A total of ten programs (six Individual and four Couples) were run in 2016 with group sizes ranging between two to eight participants. For all ten programs, the lead facilitator (and program developer) remained the same. All horses utilised across programs had prior involvement in the EAT program but the horses varied between programs, due to availability.

**Table 1:** Content for individual and couples equine-assisted therapy program.

Activity	Purpose
**Herd Meets**	Daily activity each morning to raise awareness that every day is different for self and others.
**Ground Work**	Based around learning natural horsemanship skills, these activities are undertaken daily and build in complexity and proximity to the horse.
**Grooming**	Skills based activity where participants make close and tactile contact with a horse.
**Liberty Work** **(No contact with lead rope)**	In the latter stages of the program, the participants ‘tie-off’ the lead ropes and move the horse by energetic connection only.
**Obstacle Course**	‘Metaphorical’ activity where the course represents obstacles in life.
**Trail Walks**	This activity goes from the known to the unknown, as the group takes their horses out from a fenced arena into open fields and along bush trails.
**Mindfulness**	Led mindfulness sessions are incorporated into the program. This is used to support grounding, self-regulation and provide resources for use at home.
**Photo Languages**	Undertaken at a suitable point in the program to help participants view themselves and their perspectives.
**Group Discussions**	Group discussions are led by an EEA facilitator and form a major part of the program
**Couples Dates** **(Couples Program Only)**	Each day the couple will go on a date to give the couple quality time to re-connect and share the days’ experience.
**Couples Counselling** **(Couples Program Only)**	Each couple is assigned a ‘counsellor’ from within the Facilitation team. Each day the couples will have a private session with their ‘counsellor’ to discuss their day, their date and any issues that have arisen.

#### Demographic and service information

Demographic information included age, gender, and current marital status. Veteran participants also reported service information including length and branch of service, if they were medically discharged, and years since discharge.

#### Outcome measures

Participants’ PTSD symptoms were assessed using the Posttraumatic Stress Disorder Checklist for DSM-5 (PCL-5) [37]. The PCL-5 is a 20-item self-report measure that assesses DSM-5 symptoms of PTSD. Respondents rate each item along a 5-point Likert scale from 0 (*Not at all*) to 4 (*Extremely*) according to how much each symptom affected them over the previous month. A total symptom severity score is obtained by summing responses (total scores range 0 to 80), with higher scores indicating greater severity. A provisional diagnosis of PTSD requires at least one symptom endorsed (rated 2 or above) in symptom Cluster B (items 1–5) and in Cluster C (items 6–7) and two symptoms endorsed in Cluster D (items 8–14) and in Cluster E (items 15–20). The PCL-5 has demonstrated strong psychometric properties in previous studies and is used regularly for monitoring symptom change within interventions [37–39].

Participants’ symptoms of depression, anxiety, and stress were measured using the Depression Anxiety Stress Scale– 21 (DASS-21) [40] The DASS-21 is a self-report questionnaire containing three subscales assessing the emotional states of depression, anxiety, and stress. Respondents rate each item along a 4-point Likert scale from 0 (*Did not apply to me at all*) to 3 (*Applied to me very much*, *or most of the time*) according to how much they were affected by the symptom over the previous week. Higher scores on each subscale (subscale total scores range 0 to 21) indicate greater experiences of depression, anxiety, and stress. The scale has demonstrated excellent psychometric properties in previous research [40–42].

The Oxford Happiness Questionnaire (OHQ), a 29-item self-report questionnaire, was included to assess participants’ perceived happiness [43]. Respondents rate each item along a 6-point Likert scale from 1 (*Strongly disagree*) to 6 (*Strongly agree*). Responses are summed and the total score is then divided by 29. Final scores range between 1 and 6 with higher scores indicating greater levels of happiness. Previous research has demonstrated the measure has good psychometric properties [43, 44].

Participants’ perceived quality of life was assessed using the Quality of Life, Enjoyment and Satisfaction Questionnaire–Short Form (Q-LES-Q-SF) [45]. The Q-LES-Q-SF is a 16-item self-report questionnaire that examines quality of life, enjoyment and satisfaction [45]. Respondents are asked to rate each item on a 5-point Likert scale from 1 (*Very poor*) to 5 (*Very good*) according to how satisfied they were in the item domain over the previous week. Total scores are created by summing the first 14 items (total scores range 17 to 70) with higher scores indicating greater quality of life, satisfaction and enjoyment. Previous research has indicated the questionnaire has excellent psychometric properties [46].

### Statistical analysis

All analyses were conducted using IBM SPSS 24. Independent samples *t*-tests were used to examine differences on outcome measures at pre-intervention between programs as well as completers and non-completers, and a Pearson’s chi-square test to compare drop-out rates at follow-up between programs. Due to the missing data at follow-up, repeated measures ANOVAs could not be reliably used. As such, paired samples *t*-tests were used to examine differences between pre-intervention, post-intervention, and follow-up for each program. Independent-samples *t*-tests were used to examine if significant differences were present at each time point between the programs. The assumption of homogeneity of variances was upheld for all analyses. Bonferroni correction was applied to account for multiple testing (*p* < .017) [47] and Cohen’s *d* effect sizes [48, 49] were calculated to assess the degree of change. Finally, Pearson’s chi-square were utilized to examine if rates of provisional PTSD diagnoses significantly differed at each time point between programs. Assumptions for chi-square tests were met.

## Results

### Study sample

Forty-seven participants (Individual, *n* = 25; Couples, *n* = 22) completed the equine-assisted therapy program. Demographic and service data for veteran participants are provided in Table 2. In the Couples program, partners of veterans included 1 males and 10 females aged between 31 to 57 years (Mean = 40.82, *SD* = 9.42).

**Table 2:** Demographic and service information for participants of the equine-assisted therapy programs.

	Individual Program (*n* = 25)	Couples Program (*n* = 22)
**Age in years**; Mean (*SD*), Range	50.28 (14.59), 26 to 72	42.12 (10.03), 29 to 68
**Gender**		
Male	19 (76%)	11 (10 veterans; 90.91%)
Female	6 (24%)	11 (1 veteran;9.09%)
**Current marital status**		
Married	12 (48%)	16 (72.73%)
In a relationship/De facto	3 (12%)	6 (27.27%)
Single	8 (32%)	0 (0%)
Divorced	2 (8%)	0 (0%)
**Type of service Service**		Veterans only (*n* = 11)
Airforce	3 (12%)	0 (0%)
Army	20 (80%)	9 (81.82%)
Navy	2 (8%)	2 (18.18%)
**Deployed overseas**		
Yes	16 (64%)	8 (72.73%)
No	8 (32%)	1 (9.09%)
Not reported	1 (4%)	2 (18.18%)
**Years served**; Mean (*SD*), Range	12.33 (10.77), 1 to 43	11.09 (5.28), 1 to 20
**Years since discharge**; Mean (*SD*), Range	17.92 (12.28), 2 to 48	13.55 (12.32), 4 to 47
**Medically discharged**		
Yes	10 (40%)	7 (63.64%)
No	14 (56%)	4 (36.36%)
Not reported	1 (4%)	0 (0%)

Independent samples *t*-tests revealed no significant differences between programs on any outcome measures at pre-intervention. Due to a data collection error, five participants from the Individual program did not complete the PCL-5 at pre- and post-intervention. All participants returned the other questionnaires post-intervention (100%) and 28 participants returned the PCL-5 (66.67%) and other measures (59.57%) at follow-up. Independent samples *t*-tests revealed no significant differences at pre-intervention between those who were ‘lost to follow-up’ and those who completed the follow-up questionnaires. Results of a chi-square test revealed no significant difference in ‘lost to follow-up’ rates between programs. Two participants at post-intervention and five participants at follow-up did not complete 13 or more items on the OHQ and as such, these incomplete questionnaires were removed from subsequent analyses. Table 3 displays frequency, means, and standard deviations for each measure across data collection time points.

**Table 3:** Frequency, means, and standard deviations of measures across data collection time points.

	Individual Program						Couples Program					
	Pre-Intervention		Post-Intervention		Follow-Up		Pre-Intervention		Post-Intervention		Follow-Up	
**DASS-21 Depression**	25	10.72 (5.83)	25	4.80 (4.10)	12	12.58 (6.45)	22	9.00 (6.06)	22	3.77 (3.77)	16	5.44 (2.29)
**DASS-21 Anxiety**	25	9.68 (4.89)	25	6.04 (4.26)	12	7.92 (5.05)	22	7.95 (5.82)	22	5.45 (4.00)	16	4.31 (3.46)
**DASS-21 Stress**	25	12.84 (4.84)	25	7.48 (4.79)	12	12.33 (4.77)	22	11.68 (4.91)	22	7.09 (5.09)	16	7.88 (3.85)
**PCL-5**	20	43.70 (17.82)	20	29.60 (17.90)	12	47.92 (17.51)	22	40.73 (20.27)	22	28.41 (19.75)	16	24.88 (16.57)
**OHQ**	25	3.11 (0.83)	23	3.70 (0.99)	11	3.16 (1.14)	22	3.25 (0.83)	22	3.75 (0.74)	12	3.41 (0.51)
**Q-LES-Q-SF**	25	37.88 (9.38)	25	47.88 (10.37)	12	35.92 (9.63)	22	39.86 (9.75)	22	49.64 (7.30)	16	43.31 (8.59)

DASS-21 = Depression Anxiety Stress Scale– 21. PCL-5 = Posttraumatic Stress Disorder Checklist–DSM-5. OHQ = Oxford Happiness Questionnaire. Q-LES-Q-SF = Quality of Life, Enjoyment and Satisfaction Questionnaire–Short Form.

### Within-subjects analyses

For each program, paired samples *t*-tests were used to examine if significant changes on the measures occurred between pre-intervention and post-intervention, post-intervention and follow-up, and between pre-intervention and follow-up. Table 4 displays the results of these analyses.

**Table 4:** Paired samples t-tests for individual and couples programs.

	Individual Program									Couples Program								
	Pre-Intervention– Post-Intervention			Pre-Intervention– Follow-Up			Post-Intervention– Follow-Up			Pre-Intervention– Post-Intervention			Pre-Intervention– Follow-Up			Post-Intervention– Follow-Up		
	*t*	*df*	*d*	*t*	*df*	*d*	*t*	*df*	*d*	*t*	*df*	*d*	*t*	*df*	*d*	*t*	*df*	*d*
**DASS-21 Depression**	6.31*	24	1.26	-0.32	11	-0.09	-4.00*	11	-1.16	4.43*	21	0.94	2.93*	15	0.73	-1.78	15	-0.44
**DASS-21 Anxiety**	4.58*	24	0.92	0.88	11	0.25	-1.86	11	-0.54	2.31	21	0.49	3.66*	15	0.91	1.95	15	0.49
**DASS-21 Stress**	5.47*	24	1.09	0.85	11	0.25	-3.21*	11	-0.93	3.83*	21	0.82	3.60*	15	0.90	-0.39	15	-0.10
**PCL-5**	3.92*	19	0.88	-1.52	8	-0.51	-3.49*	8	-1.16	3.14*	21	0.67	4.94*	15	1.24	1.14	15	0.29
**OHQ**	-4.35*	22	-0.91	-0.63	10	-0.19	3.58*	9	1.13	-4.24*	21	-0.90	-0.58	11	-0.17	1.26	11	0.36
**Q-LES-Q-SF**	-4.61*	24	-0.92	0.68	11	0.20	3.71*	11	1.07	-6.68*	21	-1.42	-0.77	15	-0.19	3.46*	15	0.87

DASS-21 = Depression Anxiety Stress Scale– 21. PCL-5 = Posttraumatic Stress Disorder Checklist–DSM-5. OHQ = Oxford Happiness Questionnaire. Q-LES-Q-SF = Quality of Life, Enjoyment and Satisfaction Questionnaire–Short Form.

*p < 0.017 (Bonferroni Corrected for multiple testing).

#### Individual program

For participants of the Individual program, analyses revealed significantly lower scores on the DASS-21 and PCL-5 at post-intervention compared to pre-intervention scores. Participants also reported significantly higher scores on the OHQ and Q-LES-Q-SF at post-intervention compared to pre-intervention. However, compared to post-intervention, participants reported significantly higher scores on the DASS-21 depression and stress subscales and the PCL-5 and significantly lower scores on the OHQ and Q-LES-Q-SF at follow-up. There were no significant differences between pre-intervention and follow-up on any of the measures. Effect sizes were examined and interpreted utilising Cohen’s d guidelines; *r* = 0.2 (small effect), *r* = 0.5 (medium effect), *r* = 0.8 (large effect) [48, 49]. Effect sizes for all significant differences were large.

According to interpretive guidelines of the DASS-21 [36], participants’ depression subscale scores started in the ‘Severe’ range at pre-intervention, dropped to ‘Mild’ at post-intervention, and returned to ‘Severe’ by follow-up. Anxiety scores fell within the ‘Extremely Severe’, ‘Moderate’, and ‘Severe’ ranges at pre-intervention, post-intervention and follow-up respectively. While scores on the stress subscale fell within the ‘Severe’, ‘Mild’, and ‘Moderate’ ranges at pre, post, and follow-up respectively. In terms of PTSD symptoms, the mean score on the PCL-5 reduced by greater than 10 points from pre-intervention to post-intervention, indicating a “clinically significant” change, although this change was not maintained at follow-up [50].

#### Couples program

For participants of the Couples program, analyses revealed significantly lower scores on the depression and stress DASS-21 subscales as well as the PCL-5 at post-intervention and follow-up compared to pre-intervention and significantly lower scores on the anxiety subscale at follow-up compared to pre-intervention. There was no significant difference in anxiety scores between pre-intervention and post-intervention and no significant difference in DASS-21 and PCL-5 scores between post-intervention and follow-up. Participants also reported significantly higher scores on the OHQ and Q-LES-Q-SF at post-intervention compared to pre-intervention; however, there was no significant difference between pre-intervention scores and follow-up on these measures. Additionally, participants reported significantly lower Q-LES-Q-SF scores at follow-up compared to post-intervention. Effect sizes for all significant differences were large.

According to interpretive guidelines of the DASS-21 [40], participants’ depression scores started in the ‘Moderate’ range at pre-intervention, dropped to ‘Normal’ at post-intervention and ended in the ‘Mild’ range by follow-up. Anxiety scores started within the ‘Severe’ range at pre-intervention, dropped to ‘Moderate’, at post-intervention and dropped further to ‘Mild’ by follow-up. Scores on the stress subscale fell within the ‘Moderate’, ‘Normal’, and ‘Mild’ ranges at pre, post and follow-up respectively. Additionally, from pre-intervention to post-intervention, the mean score on the PCL-5 reduced by greater than 10 points, indicating a “clinically significant” change that remained three months following the intervention [50].

### Between-groups analyses

Independent samples *t*-tests were used to compare scores on each measure, at pre-intervention, post-intervention, and follow-up, between the Individual and Couples programs (Table 5). The results of these analyses revealed no difference in baseline scores between programs, but significant differences in the depression and stress DASS-21 subscales and PCL-5 at follow-up, with participants in the Couples program reporting significantly fewer symptoms compared to participants in the Individual program. There were no additional significant differences between the programs reported. Effect sizes for significant differences were large.

**Table 5:** Independent samples *t*-Tests comparing individual and couples program scores on each measure across time points.

	Pre-Intervention			Post-Intervention			Follow-Up		
	*t*	*df*	*d*	*t*	*df*	*d*	*t*	*df*	*d*
**DASS-21 Depression**	-0.99	45	-0.29	-0.89	45	-0.26	-3.53*	26	-1.48
**DASS-21 Anxiety**	-1.11	45	-0.32	-0.48	45	-0.14	-2.24	26	-0.83
**DASS-21 Stress**	-0.81	45	-0.24	-0.27	45	-0.08	-2.74*	26	-1.03
**PCL-5**	-0.50	40	-0.16	-0.20	40	-0.06	-3.55*	26	-1.35
**OHQ**	-0.55	45	0.16	0.18	43	0.05	0.69	21	0.28
**Q-LES-Q-SF**	0.71	45	0.21	0.66	45	0.20	2.14	26	0.81

DASS-21 = Depression Anxiety Stress Scale– 21. PCL-5 = Posttraumatic Stress Disorder Checklist–DSM-5. OHQ = Oxford Happiness Questionnaire. Q-LES-Q-SF = Quality of Life, Enjoyment and Satisfaction Questionnaire–Short Form.

*p < 0.017 (Bonferroni Corrected for multiple testing).

**Fig 1:**
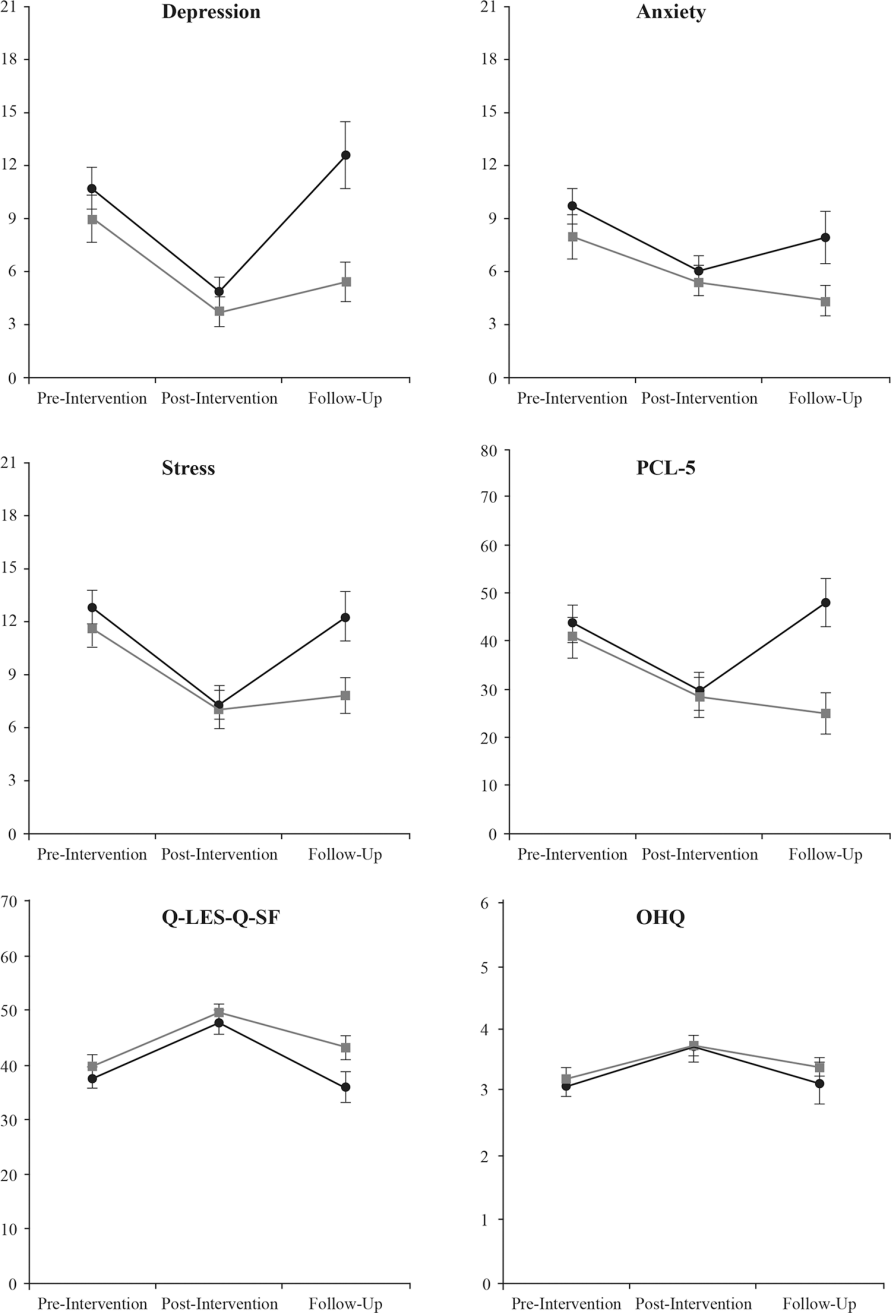
Mean scores on outcome measures at pre-intervention, post-intervention, and follow-up for each program. Grey line represents Couples Program. Black line represents Individual program. Error bars represent standard errors.

Chi-square tests were utilized to determine if there were significant differences in frequency of provisional PTSD diagnoses between programs at pre- intervention, post-intervention, and follow-up (Table 6). Results revealed no significant differences between programs at pre-intervention and post-intervention. There was a significant difference at follow-up with fewer participants in the Couples program meeting criteria for provisional PTSD diagnosis compared to participants in the Individual program. Fig 1 represents mean scores on outcome measures at pre-intervention, post-intervention, and follow-up for each program.

**Table 6:** Chi-square tests comparing provisional PTSD diagnoses between programs at each time point.

	PTSD Diagnosis	No PTSD Diagnosis		
**Program**	*n* (%)	*n* (%)	χ^2^	*p*
***Pre-Intervention***				
**Individual**	15 (37.7)	5 (11.9)	0.24	.625
**Couples**	15 (37.7)	7 (16.7)		
***Post-Intervention***				
**Individual**	9 (21.4)	11 (26.2)	0.11	.746
**Couples**	11 (26.2)	11 (26.2)		
***Follow-Up***				
**Individual**	10 (35.7)	2 (7.1)	7.48	.006
**Couples**	5 (17.9)	11 (39.3)		

PTSD = Posttraumatic stress disorder.

## Discussion

This study aimed to evaluate the psychological outcomes of an equine-assisted therapy program for veterans and their partners, specifically across the domains of depression, anxiety, stress, posttraumatic stress, happiness, and quality of life as well as compare the outcomes of an Individual and Couples program. Given the limited research and methodological limitations in the existing literature examining the utility of equine-assisted therapy within military and veteran populations, this research contributes to the international literature through the inclusion of a couples condition as well as follow-up assessment.

The analyses of the Individual program indicated that symptoms of depression, anxiety, stress, and PTSD significantly reduced and participants’ self-reported happiness and quality of life significantly increased from the beginning of the program to the conclusion of the program. However, results also demonstrated these gains were short-term, with scores on all measures, except anxiety, returning to pre-intervention levels three months following the conclusion of the program. Analyses of the Couples program indicated that symptoms of depression, stress, and PTSD significantly reduced by the conclusion of the program and this reduction remained three months later. The analysis also demonstrated a gradual reduction in anxiety symptoms from pre-intervention resulting in a significant reduction at the three month follow-up point. Participants’ self-reported happiness and quality of life significantly increased from the beginning to the conclusion of the program, although, this increase did not remain three months later. The results of the Couples program are in line with previous findings suggesting some benefits exist for the use of equine-assisted therapy with veterans with mental health difficulties [25, 33].

When comparing participants’ outcomes in the Individual and Couples programs, both veterans and partners who completed the Couples program reported significantly less symptoms of depression, stress, and PTSD three months following the conclusion of the program, despite no difference prior to commencing the program or at post-intervention. In addition, significantly fewer participants in the Couples program met provisional diagnosis of PTSD at follow-up compared to participants of the Individual program. Further, the clinically significantly reduction in PTSD symptoms noted at post-intervention only remained three months later for participants of the Couples program. It could be possible that the Couples program may facilitate greater psychological outcomes long-term than the Individual program. As the Couples program includes involvement of the veteran’s partner, this perhaps indicates that adaptive coping strategies developed during the program can be rehearsed and reinforced with the partner once the program concludes, leading to ongoing stability of psychological symptoms. This is in line with previous literature, which suggests involvement of a family member in therapy may be beneficial for veterans [51, 52]. The Couples program also included additional couples therapy strategies within the intervention. As such, the difference may also be influenced by the improved quality of the intimate relationship, which has been associated with better mental health generally in previous research [53, 54]. It may also be possible that veterans with partners willing to engage in therapy are likely more supportive which may lead to better long-term outcomes for the veteran, independent of type of intervention.

### Limitations

Despite the strengths of the research, there are limitations that must be acknowledged. First, as this was an evaluation of an existing service, the study design did not include a control group. As such, conclusions regarding efficacy of the intervention cannot be made as outcomes could be attributed to other non-controlled factors including participation in other therapeutic activities. Additionally, the sample size for quantitative data analysis remained small and as such, the type of analysis that could be conducted was restricted. In particular, the impact of the dyadic data on the outcomes was not able to be assessed using multi-level modelling, and analysis of variance between the 10 programs was not completed. Finally, a substantial proportion of participants were lost to follow-up at three months. This further restricts the interpretation of the findings.

### Future research and practical implications

To determine the utility of equine-assisted therapy within a veteran population with more certainty, future research should proceed in a number of ways. Conducting a wait-list controlled trial of the equine-assisted therapy program, increasing the sample size, and improving data collection procedures at follow-up to improve response rates would be of use. Using a mixed methods approach and collecting both qualitative and quantitative data may also be useful in determining effectiveness of a program, as well as providing greater insight regarding the mechanisms of change of the intervention. Finally, including an assessment of relationship quality would also be useful in the future, and may help determine if there is a mediating role of relationship quality between the Couples program and improved mental health. In terms of practical implications for the field, this study generates some questions regarding the durability of treatment gains of a five day EAT program. This means that EAT program developers and clinicians may need to carefully consider the length of the intervention being delivered and to ensure a method of follow-up is included, to mitigate potential mental health deterioration among participants. This may include the implementation of EAT ‘booster sessions’ which occur at regular intervals following core program conclusion, or referral to a mental health provider at the end of the program to facilitate ongoing self-reflection and consolidation of skills learnt. However, robust recommendations cannot be made until findings of this study are replicated with the above limitations addressed.

## Conclusions

Given the limited research examining the utility of equine-assisted therapy within veteran populations, this research contributes to the international literature by assessing the psychological outcomes of an EAT program using quantitative psychometric measures, comparing the outcomes of both a Couples and an Individual EAT program, and by including follow up assessment, 3 months after the program. The results indicate that equine-assisted therapy might be useful in the reduction of depression, anxiety, stress, PTSD symptoms and the improvement of happiness and quality of life, but that these gains may only be short-term unless partners are integrated into the intervention. While these findings demonstrate a promising trend, no conclusions regarding efficacy can be made and a controlled trial, with a larger sample size would help determine if equine-assisted therapy is an effective adjunct intervention for veterans.
